# Longitudinal monitoring of IL-6 and CRP in inflammatory bowel disease using IBD-AWARE

**DOI:** 10.1016/j.biosx.2023.100435

**Published:** 2024-01-06

**Authors:** Robert P. Hirten, Kai-Chun Lin, Jessica Whang, Sarah Shahub, Nathan K.M. Churcher, Drew Helmus, Sriram Muthukumar, Bruce Sands, Shalini Prasad

**Affiliations:** a The Dr. Henry D. Janowitz Division of Gastroenterology, Icahn School of Medicine at Mount Sinai, New York, NY, USA; b Department of Bioengineering Engineering, The University of Texas at Dallas, Richardson, TX, USA; c EnLiSense LLC, Allen, TX, USA

**Keywords:** Wearable devices, Inflammatory bowel disease (IBD), Cytokines, CRP, IL-6 sweat-based sensor

## Abstract

There are limitations to monitoring modalities for chronic inflammatory conditions, including inflammatory bowel disease (IBD). Wearable devices are scalable mobile health technology that present an opportunity to monitor markers that have been linked to worsening, chronic inflammatory conditions and enable remote monitoring. In this research article, we evaluate and demonstrate a proof-of-concept wearable device to longitudinally monitor inflammatory and immune markers linked to IBD disease activity in sweat compared to expression in serum. Sixteen participants with an IBD-related hospital admission and a C-reactive protein (CRP) > 5 μg/mL were followed for up to 5 days. The sweat sensing device also known as IBD AWARE was worn to continuously measure CRP and interleukin-6 (IL-6) in the sweat of participants via electrochemical impedance spectroscopy. Serum samples were collected daily. A linear relationship between serum and sweat readings for CRP and IL-6 was demonstrated based on individual linear correlation coefficients. Pooled CRP and IL-6 serum-to-sweat ratios demonstrated improving correlation coefficients as serum cutoffs decreased. Between the first and last day of observation, significant and non-significant trends in serum CRP and IL-6 were observed in the sweat. Comparison of sweat measurements between the subjects with active IBD and 10 healthy subjects distinguished an inflamed and uninflamed state with an AUC of 0.85 (95% CI: 0.68–1.00) and a sensitivity and specificity of 82% and 70% at a CRP cutoff of 938.9 pg/mL. IBD AWARE wearable device holds promise in longitudinally monitoring individuals with IBD and other inflammatory diseases.

## 1. Introduction

Inflammatory bowel disease (IBD), comprised of Crohn’s disease (CD) and ulcerative colitis (UC), are relapsing, and remitting chronic inflammatory conditions of the gastrointestinal tract (Torres et al., 2017; Ungaro et al., 2017). It is characterized by symptoms including abdominal pain, diarrhea, and blood per rectum. In CD, any part of the gastrointestinal tract from the mouth to the anus can be chronically inflamed whereas in UC, the chronic inflammation is usually confined to the colon (large intestine) and the rectum (Baker and Gore, 2015; Torres et al., 2017; Ungaro et al., 2017). While its etiology is not fully understood, research points to a combination of genetic, immunoregulatory and environmental factors as the triggers for this abnormal autoimmune disease (Baumgart and Carding, 2007; “Inflammatory Bowel Disease (IBD) | Johns Hopkins Medicine,” n.d.; “Inflammatory bowel disease (IBD) - Symptoms and causes - Mayo Clinic,” n.d.; “What is inflammatory bowel disease (IBD)? | IBD,” n.d.). When the body is infected, as a defense mechanism, the body ramps up the attack on the affected areas in a process called inflammation that can exhibit symptoms such as swelling, pain or bruising (Ciaccia, 2011). In healthy patients, once a threat is neutralized by the body’s immune system, the inflammation processes ramp down to return the body to a normal uninflamed state. Conversely, in IBD patients, there is no taming of the gut inflammation once triggered until there is external intervention (Hanauer, n.d.). The condition is challenging due to discordant symptoms and inflammation and unpredictable flares (Bolge et al., 2010; Colombel et al., 2017). The CDC reports that as many as 3 million of adults in the U.S. were diagnosed in 2015 and the prevalence of other chronic comorbidities such as arthritis increases with patients with IBD (“What is inflammatory bowel disease (IBD)? | IBD,” n.d.).

Management has shifted to a treat-to-target approach which aims to achieve both clinical remission and mucosal healing, necessitating an understanding of inflammatory activity (Colombel et al., 2017). There are important limitations to currently used modalities to monitor inflammation in IBD. Currently, the means of diagnosis and monitoring of IBD are classified into two main techniques- Laboratory tests and Imaging/endoscopy (“English | World Gastroenterology Organisation,” n.d.). Under the laboratory tests, they range from blood tests for inflammation markers such as C-reactive protein (CRP) to stool examination for inflammation markers such as calprotectin. A combination of imaging techniques such as contrast radiography and endoscopy methods such as ileocolonoscopy remain the gold standard for diagnosis of IBD (“English | World Gastroenterology Organisation,” n.d.; “Inflammatory Bowel Disease (IBD) | Johns Hopkins Medicine,” n.d.; “What is inflammatory bowel disease (IBD)? | IBD,” n.d.). However, they are all invasive, limited to a single time point and are inconvenient to collect (Mosli et al., 2015). This underscores the need to develop new methods to monitor inflammatory activity for real-time, actionable determination of medication response and disease status.

Wearable technology is increasingly used and accepted by patients with IBD (Hirten et al., 2021b). These devices can monitor physiological parameters both non-invasively and continuously (Hirten et al., 2021a). This provides an opportunity for improved monitoring of chronic diseases such as IBD. Sweat is a biofluid that contains analytes including proteins, such as cytokines and inflammatory markers, that are easily and non-invasively accessible. Prior work has demonstrated that markers in the sweat and serum are similar in healthy subjects making this an attractive area of exploration (Cizza et al., 2008). Our group has developed a novel wearable device that can continuously monitor sweat analytes in real-time. Leveraging this technology, we previously demonstrated its ability to measure interleukin (IL)-1 beta (β) and CRP in healthy controls (Jagannath et al., 2020).

Cytokines are chemical messengers important for cell signaling and the regulation of inflammation. They play a key role in IBD, with an imbalance between pro and anti-inflammatory cytokines impacting inflammation development, recurrence, and exacerbation (Sanchez-Muñoz et al., 2008). IL-6 relates to IBD pathophysiology and correlates with mucosal inflammation (Al-Sadi et al., 2008). Along with CRP, an inflammatory marker that tracks inflammatory activity, inflammatory and immune activity markers are attractive targets for non-invasive continual monitoring of disease activity (Solem et al., n.d.). Usually, blood is the primary biofluid used in the monitoring of these markers, but it is invasive and not amenable to continuous or frequent assessments. Being able to understand the correlation between the CRP and IL-6 in serum and sweat in IBD patients would provide a unique opportunity to monitor IBD for flare ups non-invasively.

**Fig. 1. |:**
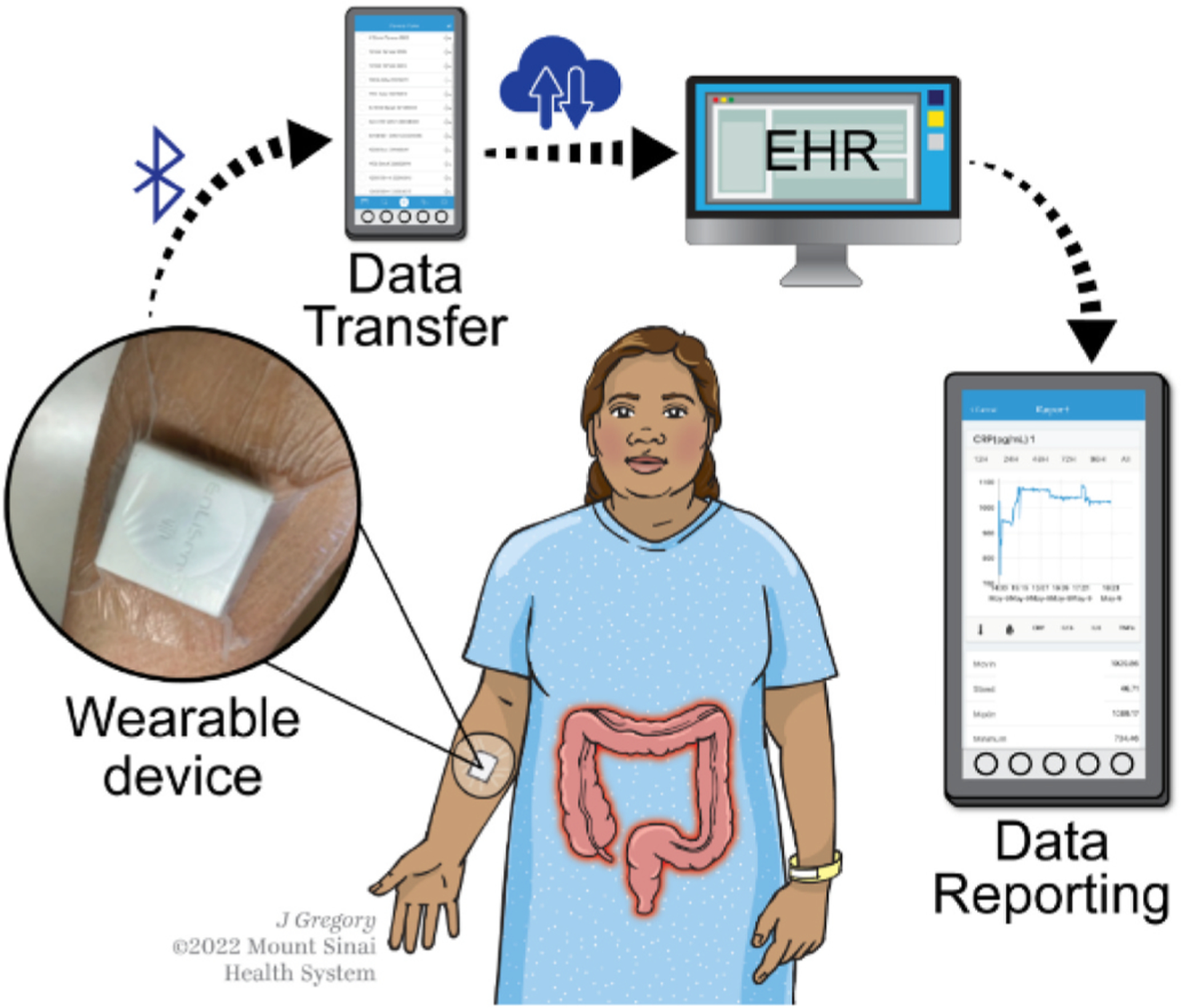
The IBD AWARE device collects near-continuous readings of protein analytes in the sweat. This data is transferred via Bluetooth through a smart-phone or tablet to the cloud server. Data can be visualized by healthcare providers or patients via a custom portal or via integration with electronic health systems. This provides real-time disease state assessment. **EHR**- Electronic Health Record.

Our IBD AWARE device is modifiable and able to measure up to 4 sweat analytes at a time. Building on our prior work with our novel sweat-sensing device we launched the current study to perform the first longitudinal assessment of the relationship between cytokines and inflammatory markers in the sweat and serum and the first evaluation of their relationship in a chronic inflammatory disease (Fig. 1). This pilot study is a proof of concept for the exploration of other sweat cytokines and inflammatory markers in IBD, as well as other chronic inflammatory diseases.

## 2. Materials and methods

### 2.1. Study design

Patients with IBD were enrolled in a prospective observational pilot study at The Mount Sinai Hospital, New York. Eligible participants were aged ≥18 years, diagnosed with either CD or UC, admitted to the Mount Sinai Hospital for an IBD-related admission and had an elevated CRP (>5 μg/mL) at the time of enrollment. Participants were excluded if they had an active infection, were planning to undergo surgery during the admission, had a bowel obstruction, were not willing to wear the sweat-sensing device, or have blood drawn as part of the study. A separate cohort of healthy control subjects without IBD or other health issues were enrolled at the University of Texas at Dallas, Dallas, Texas. The subjects were aged 16–75 years and were excluded if they had an active infection or chronic medical condition. This study was approved by the institutional review boards at Mount Sinai and the University of Texas at Dallas (IRB number UTD IRB 19–146), respectively. All subjects signed an informed consent.

### 2.2. Study procedures

Baseline demographic information, IBD history and medication usage were collected at enrollment in the IBD cohort. IBD-related medications administered during the hospital admission were captured each day. Daily laboratory studies were collected assessing CRP and IL-6. Subjects were followed for up to 5 days or until hospital discharge, if sooner. An IBD AWARE device was applied to the ante-brachial region of each subject’s arm and left in place for 24 h, at which time data were synchronized to the cloud server and replaced with a fully charged device. On-body measurements were obtained every 1-min for each 24-h period, with concentration profiles reported over the recording period.

### 2.3. IBD AWARE device and immunoassay

The IBD AWARE device comprises a plastic reader and a replaceable polymer sweat-sensing strip with zinc oxide (ZnO) coated electrodes. It is manufactured through a screen-printing technique that allows for an affinity-based interaction between a capture probe antibody and the target molecule generating electrochemical activity (Munje et al., 2015). 5 μL of 30 μg/mL of monoclonal IL-6 or CRP capture antibody is immobilized on the strip via a cross-linker. 10 mM of the thiol cross-linker is immobilized via thiol bonds to the ZnO electrodes on one side and the other end binds to the capture antibody. The CRP and IL-6 molecules then bind specifically to the immobilized antibody to create the immunoassay. To capture the binding events happening at the electrode-electrolyte interface, non-faradaic electrochemical impedance spectroscopy (nf-EIS) was employed to assess the response of the IBD AWARE device.

A low sinusoidal input voltage is swept across the working electrode over a frequency of 100–1000 Hz to generate an impedance spectrum as a result of the binding of the target biomarker with the antibody on the capture probe (Lin et al., 2017). The impedance spectrum is analyzed to build a dose response curve correlating the change in impedance to a dose concentration of CRP/IL-6. This allows for the quantification of unknown concentrations of CRP/IL-6 given a known impedance change. When the IBD device is placed on the arm of the patient, sweat eluted is captured by the sweat sensing strip. CRP or IL-6 present in the sweat bind specifically to their respective immobilized antibodies. Impedance measurements are then taken and compared to established dose response curves to quantify the CRP/IL-6 in the sweat.

### 2.4. Sweat processing

Sweat CRP and IL-6 on-body measurements were obtained every 1-min for each 24-h period. The recorded measurements were averaged for every consecutive 2-h period from the start of each collection period and plotted as the temporal profile of each marker by day. The average measured concentration over the total 24-h period of each day was also calculated and compared to the concentration measured from serum collected on the same day.

### 2.5. Statistical analysis

Statistical analysis and data plotting were performed using GraphPad Prism version 9.3.0 software. Serum and sweat correlation coefficient for CRP and IL-6 was determined with the linear correlation test for each individual subject. The mean serum-to-sweat ratio was calculated across the patient cohorts for CRP and IL-6, respectively, and the coefficient of variation of the serum-sweat ratios was calculated for each cohort. Correlation coefficients were also calculated using an aggregate of the total CRP and IL-6 data set, the CRP, and IL-6 data equal to or less than 20 μg/mL and 20 pg/mL, respectively, and the CRP and IL-6 data equal to or less than 10 μg/mL and 10 pg/mL, respectively. These cut-offs were chosen to provide assessment of the dataset at decreasing analyte concentrations. This allows for the exploration of correlations at various serum-sweat levels and to evaluate for the potential saturation of sweat at different analyte levels. Two-tailed Wilcoxon signed rank test (α = 0.05) was used to determine whether the sweat and serum CRP and IL-6 concentration of the first and last day of monitoring belonged to significantly different populations. A Mann-Whitney *U* test was used to compare 24-h sweat CRP and IL-6 values derived from subjects with IBD and healthy controls. Performance was assessed with an Area Under the Receiver Operating Characteristic (auROC) curve, sensitivity, and specificity.

## 3. Results

### 3.1. Demographics

16 participants were enrolled between November 2021 and August 2022, when data were censored for analysis (Table 1). The median age at enrollment was 41.5 years, and 62.5% of participants (10/16) were women. Ten subjects had UC, 5 subjects had CD and 1 subject had indeterminate colitis. 43.8% of subjects were on a biologic agent at enrollment. Subjects were followed for a median of 4 days. Ten healthy subjects were enrolled in a separate cohort. The median age at enrollment of 26 years, and 30% of participants (3/10) were women (Supplemental Table 1).

### 3.2. Correlation of serum and sweat metrics

**Fig. 2.**
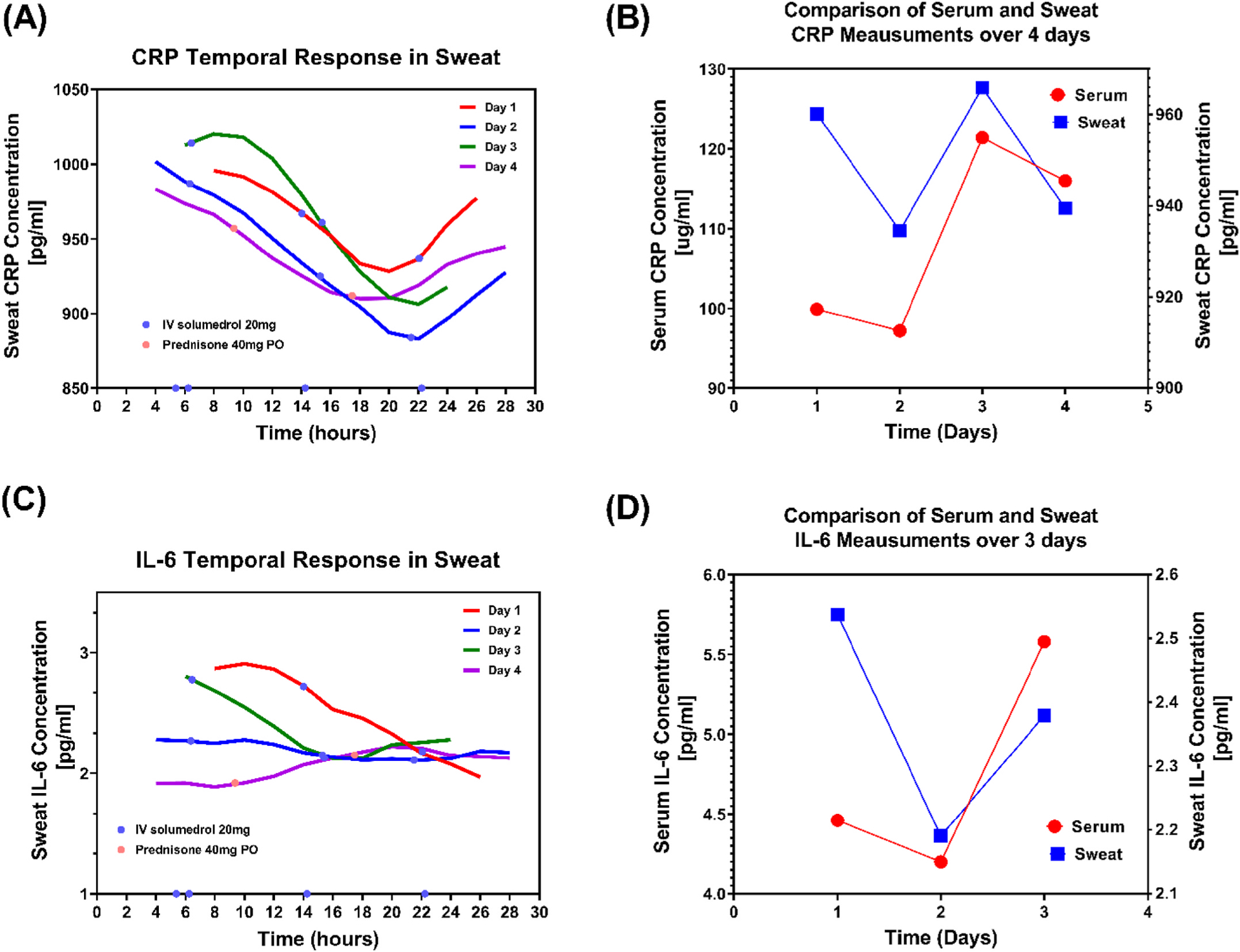
Temporal Response of (A) CRP and (C) IL-6 from the IBD AWARE device across 4 days of stay in hospital with superimposed interventions. (B) and (D) illustrate the comparison of serum and 24- hour averaged sweat measurement trends of CRP and IL-6 across 4 days of measurement. (A), (B), (C) and (D) are measured from a participant with Crohn’s disease.

The IBD AWARE device demonstrated the ability to measure sweat CRP in the range of 525–1175.38 pg/mL and IL-6 in the range of 0.45–3.14 pg/mL as highlighted in Supplemental Table 2. Fig. 2 shows the comparison between the longitudinal temporal sweat data collected from the IBD AWARE device and the serum data obtained from lab-based methods. Fig. 2a and c demonstrate the longitudinal temporal response of sweat CRP and IL-6 data collected from the IBD AWARE device in a subject over a 4-day period. A comparison between averaged 24-hr sweat measurements and daily serum measurements over 4 days showed a similarity in trend (the rise and fall) in both CRP (Fig. 2b) and IL-6 (Fig. 2d).

Simple linear regression was used to compare longitudinal serum and sweat levels. Subjects with two or more serum readings that overlapped by at least 2 h with sweat measurements, were included in the analysis. The serum-to-sweat ratio of CRP was plotted for each of the 8 subjects (Supplemental Figure 1). The coefficient of determination (R^2^) was calculated for each subject (Table 2). The mean CRP serum-to-sweat ratio was 335,955.25 with a coefficient of variation of 15.1%. IL-6 serum and sweat ratios were also plotted for each of the 6 subjects (Supplemental Figure 2). The correlation coefficient was calculated for each subject (Table 2). The mean IL-6 serum-to-sweat ratio was 36.27 (std = 6.31). The coefficient of variation was 17.4%. Two additional subjects had an overlap of serum and sweat CRP, while one additional subject had an overlap of IL-6 readings. However, at least one of the numerical results for each of these subject’s sweat sensor readings were out of the sensor’s reliable reporting range and could not be incorporated in the analysis.

**Fig. 3.**
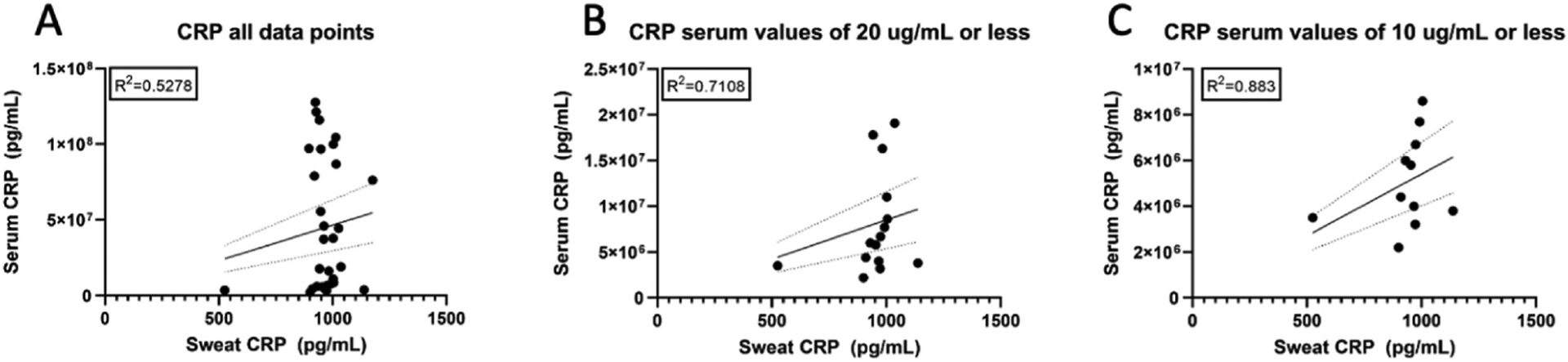
Correlation between CRP measured in serum (lab-based) and sweat (from the IBD AWARE device) for (A) the full data set of CRP values, (B) serum CRP values of ≤20 μg/mL, (C) serum CRP values of ≤10 μg/mL (C).

The serum-to-sweat ratios of CRP were plotted together for all 11 subjects who had at least one serum and sweat CRP value that overlapped by at least 2 h. The R^2^ was calculated for the overall data set (R^2^ = 0.5278) (Fig. 3a). Interestingly, visual inspection of this scatter plot demonstrates a sweat CRP threshold of approximately 880–910 pg/mL, beyond which higher serum CRP values do not translate to an appreciable rise in sweat CRP. Therefore, correlations between sweat and serum readings were evaluated at lower serum values of CRP. The correlation coefficient was calculated for all serum CRP values that were equal to or less than 20 μg/mL (R^2^ = 0.7108) (Fig. 3b). Serum CRP values that were equal to or less than 10 μg/mL had an R^2^ = 0.883 (Fig. 3c).

**Fig. 4.**
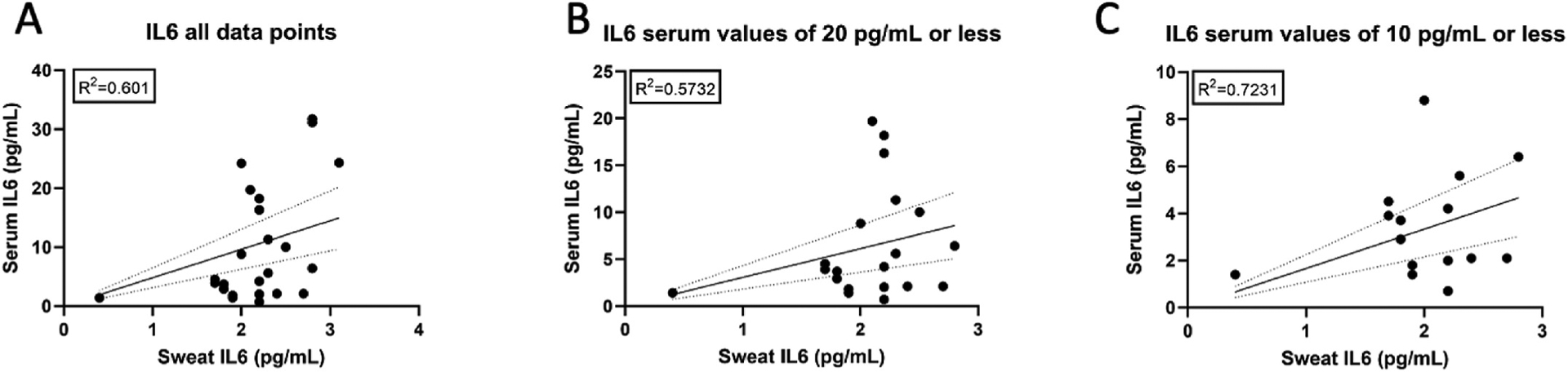
Correlation between IL-6 measured in serum (lab-based) and sweat (from the IBD AWARE device) for (A) the full data set of IL-6 values, (B) serum IL-6 values of ≤20 pg/mL, (C) serum IL-6 values of ≤10 pg/mL (C).

The serum-to-sweat ratios of IL-6 were plotted together for all 11 subjects with at least one serum and sweat IL-6 value that overlapped by at least 2 h. The overall dataset demonstrated a correlation coefficient of R^2^ = 0.601 (Fig. 4a). As with CRP, we evaluated lower serum thresholds to see if the sweat-to-serum correlation is improved toward the lower range of values. The correlation coefficient was calculated for all serum IL-6 values that were less than or equal to 20 pg/mL (R^2^ = 0.5732) (Fig. 4b). Serum IL-6 values that were less than or equal to 10 pg/mL had an R^2^ = 0.7231 (Fig. 4c).

### 3.3. Relational analysis in serum and sweat metrics

**Fig. 5.**
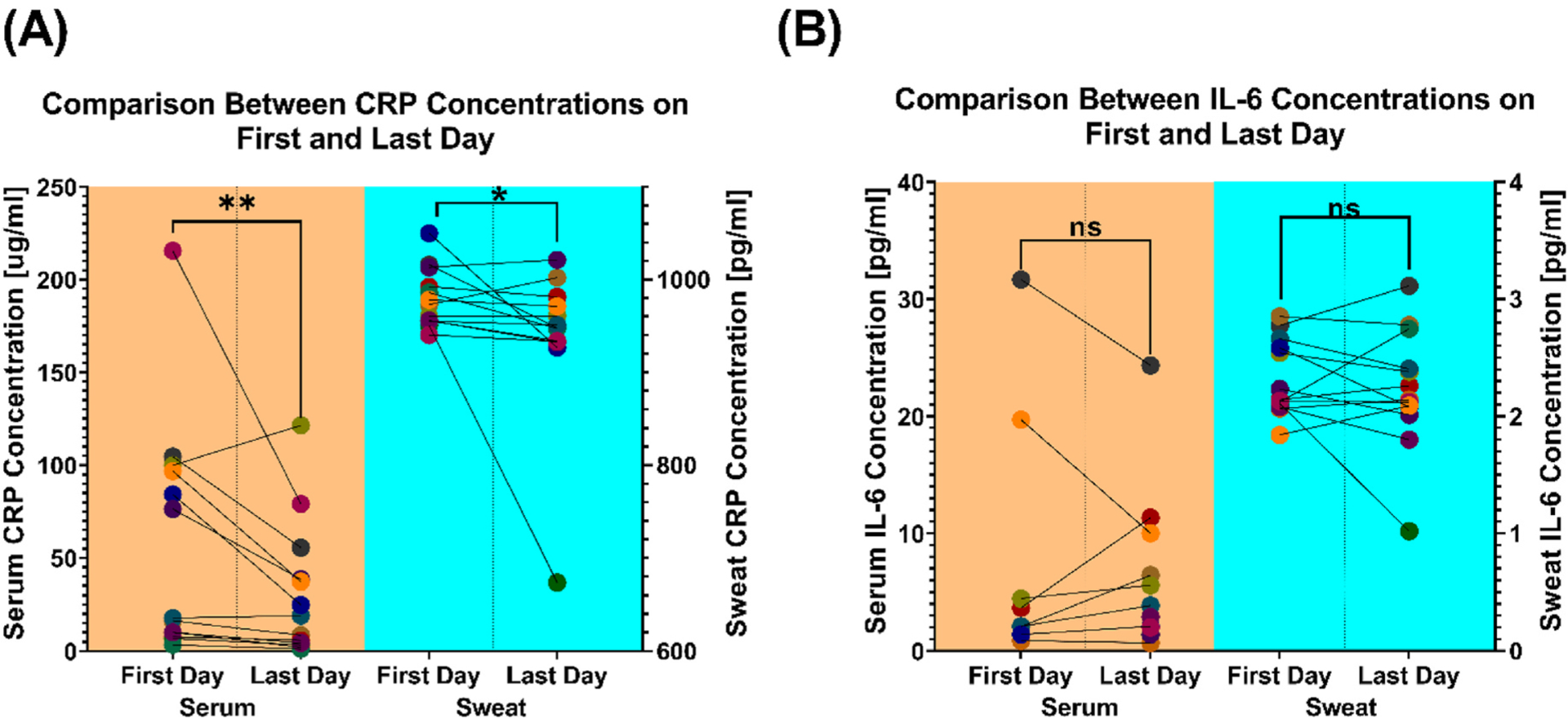
Changes in serum and sweat analytes between the first and last day of enrollment for CRP (A) and IL-6 (B). Statistical analysis for significance between the first and last day measurements for both CRP and IL-6 was computed with the Wilcoxon Test: **p = 0.0081, *p = 0.0327.

Relational analysis in serum and sweat analytes, between the first and last day of enrollment, was performed. The goal of this analysis was to determine whether the trends were mirrored between the two bio-fluids, i.e., sweat and serum. Sweat metrics were averaged over a 24-h period. A significant change in serum (p = 0.0081) and sweat (p = 0.0327) CRP was observed over the observation period (Fig. 5a). IL-6 did not demonstrate a significant change between the first and last day of enrollment in the serum (p = 0.7422) and sweat (p = 0.5417) (Fig. 5b). Given the variability in the half-life of inflammatory analytes in the sweat and serum, we analyzed sweat values over different time periods around the serum point estimate (−2 h, ± 20 min,±10 min, ± 5 min) (Supplemental Table 3 and Supplementary Figure 3). We did not find the significant and non-significant changes in the serum to be reflected in the changes in the sweat measurements for any of these other time periods.

### 3.4. Clinical classification

**Fig. 6.**
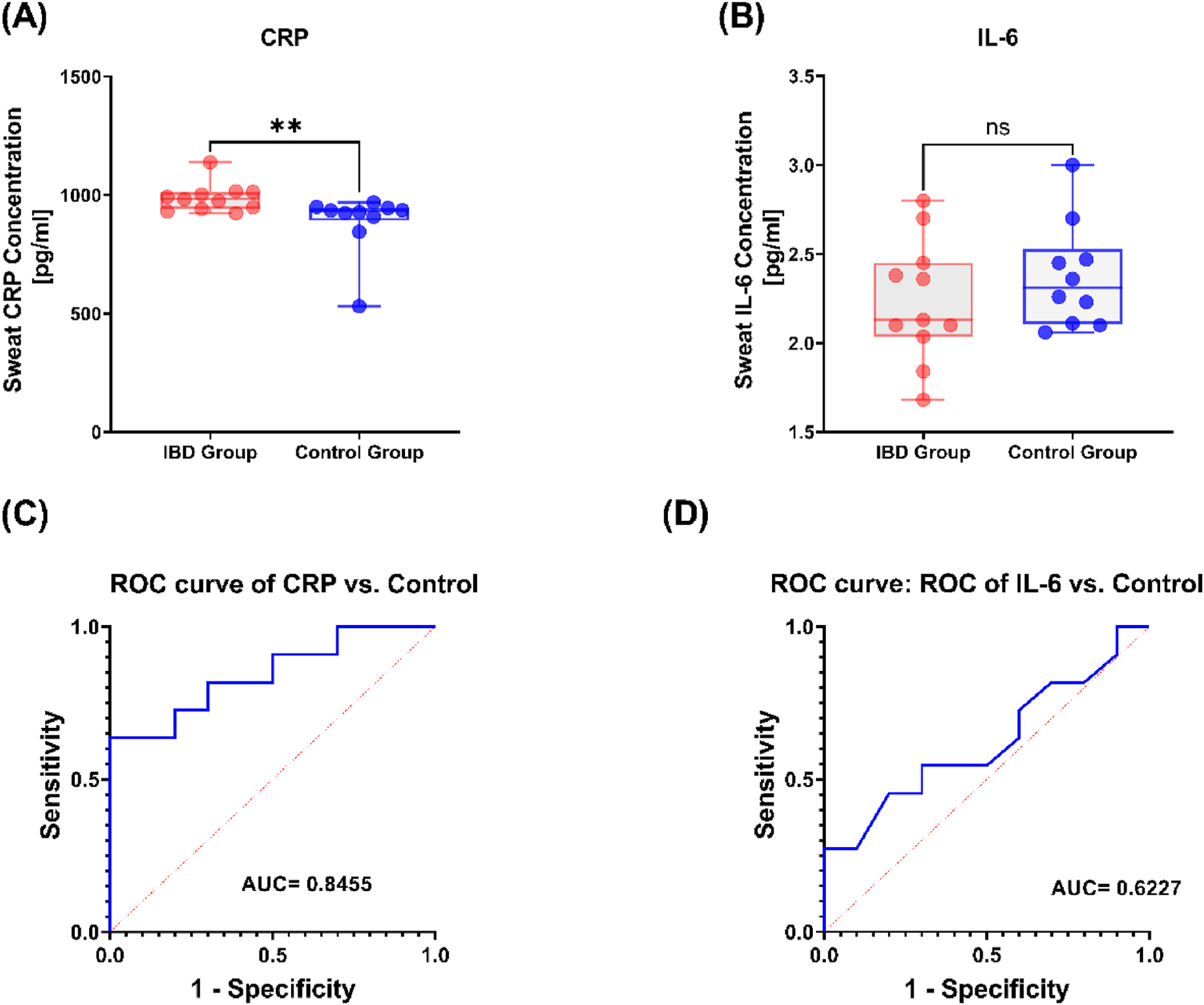
Classification of IBD patients from control group from sweat measurements of (A) CRP and (B) IL-6. Statistical analysis for significance between the healthy and inflamed group was computed with the Mann- Whitney *U* test: **p = 0.0062. Receiver Operating Characteristic (ROC) curves for (C) CRP and (D) IL-6 with computed areas under the curve (AUC) displayed at the bottom right of the respective graphs.

The measurements of the CRP and IL-6 via the IBD AWARE device were assessed to determine its utility in the classification of individuals with active inflammation (i.e., active IBD) versus no inflammation (i.e., healthy individuals). The first day of sweat CRP and IL-6 measurements were obtained from individuals in the IBD cohort and compared to first day measurements collected in a control group of 10 healthy subjects. In Fig. 6a, it can be observed that the IBD AWARE device is able to distinguish between the healthy uninflamed control group from the inflamed IBD patients based on the measured CRP (IBD group: 987.6 pg/mL [std = 59.52], uninflamed control group: 894.1 pg/mL [std = 133.7]) in sweat (p = 0.0062). In agreement with the above results, there is no significant difference between the levels of IL-6 (IBD group: 2.234 pg/mL [std = 0.34], uninflamed control group: 2.349 pg/mL [std = 0.30]) measured in sweat with the IBD AWARE device for the IBD group compared to the control group (Fig. 6b).

To further buttress this point, the Receiver Operating Curve (ROC) curve was plotted for both CRP and IL-6 as shown in Fig. 6c and d respectively. The ROC gives information about the area under the curve (AUC) which is used to determine the ability to distinguish between the inflamed IBD and uninflamed control groups. It also gives information about the sensitivity (detecting subjects with inflammation and IBD) and selectivity (ability to identify the uninflamed control as non-IBD patients). In agreement with the significant difference observed in Fig. 6a, we observe an AUC of 0.8455 (95 % CI: 0.6772 to 1.000) and a sensitivity of selectivity of 82% and specificity of 70% at a 938.9 pg/mL cutoff for CRP. As expected with IL-6, an AUC of 0.62 was computed with a sensitivity of 54%.

## 4. Discussion

In this prospective cohort study, longitudinally collected cytokines and CRP, measured in the sweat via the IBD AWARE device, correlated with serum measurements in individual subjects with IBD. To understand whether sweat and serum measurements longitudinally track across several days, first visual comparisons between the trends of both CRP and IL-6 were made. In Fig. 2, we demonstrate the tracking of serum and 24 h averaged sweat data trends over the study period. Analysis of the pooled values revealed that as the serum values of CRP and IL-6 decreased, the correlation between sweat and serum measurements increased. Furthermore, significant, and non-significant changes over time in serum values were reflected in the observed changes in the sweat. To the best of our knowledge this is the first study to show a relationship between cytokines and inflammatory markers in the serum and sweat over time and in a chronic disease state. Taken together, this pilot study identifies a possible novel means of monitoring IBD activity using non-invasive wearable technology for real-time and continuous assessment.

In addition to invasive or burdensome testing, such as ileocolonoscopy or enterography, current means of monitoring IBD activity between visits rely on the periodic assessment of inflammatory markers such as CRP and fecal calprotectin (Mosli et al., 2015). Unfortunately, such tests are limited to a single point in time, require a laboratory visit and can vary throughout the day and between days. Advances in digital technology offer a unique opportunity to improve disease management by providing frequent real-time assessments that fill in the data gaps generated by traditional monitoring modalities. Such devices offer an advantage over laboratory testing and app-based platforms through their passive nature. Wearable devices are commonly used by the general population and by individuals with chronic diseases such as IBD (“21% of Americans use a smart watch or fitness tracker | Pew Research Center,” n.d.). Large survey studies have demonstrated that patients with IBD strongly believe that wearable devices can provide important information about their health, with over 90% of individuals willing to use such a device if it could assist them in better managing their disease (Hirten et al., 2021c). While commercially available devices contain optical sensors that assess physiological parameters such as heart rate, heart rate variability and physical activity, they are not able to directly assess inflammatory activity.

Sweat offers a unique substrate that can be sampled non-invasively and continuously. The measurements of analytes in the sweat offer a novel means to assess inflammation and cytokine levels. Cizza et al. previously showed that levels of IL-6 and TNF-α in the sweat and blood are similar in healthy people 11. Our group built on this observation by employing a novel sweat sensing device in healthy subjects, demonstrating the ability to measure CRP in the sweat, as well as track CRP and IL-1β levels for over 30 h in healthy controls (Jagannath et al., 2020). However, how these analytes change over time and whether the relationship between serum and sweat-based metrics correlate over time was not known. Our current study is the first application of sweat-sensing wearable technology for the longitudinal monitoring of a chronic disease and the first to evaluate their correlation with serum levels. Our findings demonstrate a correlation between serum and sweat measurements of CRP and IL-6, with a coefficient of variation of less than 20% for each analyte. Furthermore, our finding that significant and non-significant changes in serum CRP and IL-6 are mirrored by similar significant and non-significant changes in 24-h sweat measurements demonstrates that meaningful fluctuations in serum analytes are reflected in the sweat. As an exploratory analysis, we analyzed sweat values obtained during different time periods around the time of serum collection. We did not find that the significant and non-significant changes in the serum were reflected in the sweat measurements over these other time frames. It is not clear why there is a lack of concordance between significant and non-significant changes in the serum and sweat over time, when evaluated at shorter sampling periods. One possible explanation may be that rapid fluctuations in sweat measurements observed during these shorter observation periods don’t reflect the overall trend of the analyte over the several days of observation. Additionally, further understanding is needed of the latency period between how long it takes for changes in serum analyte concentrations to be reflected in the sweat. A delay in uptake to the sweat, outside the shorter sampling window, might contribute to such findings.

Interestingly, we noted that as serum cytokine and inflammatory marker levels increased further from the normal range, correlations between sweat and serum markers weakened. This was particularly evident for CRP, which was noted to reach a sweat saturation point at approximately 880–910 pg/mL, with few values exceeding this range. Serum proteins are believed to enter sweat passively via an osmotic gradient with the serum. Our observation demonstrates that some serum analytes may reach an upper limit in the sweat and may be more evident for CRP given its relatively large molecular size (Moutachakkir et al., 2017). However, further study of this observation is needed as well as an exploration of whether such findings are evident in other analytes. This observation is not a limitation of the sweat sensing technology, which we have shown has a large range of detection (Jagannath et al., 2022). Despite this finding, strong correlations exist between sweat and serum at important physiological ranges for disease monitoring. For example, sweat and serum CRP have strong correlations below 20 μg/mL. It should be noted that the serum cutoffs were chosen to evaluate the sweat-to-serum correlations at decreasing concentrations, and not because of their clinical significance. Future studies, in larger datasets, can explore these correlations at more frequent cutoffs to understand the saturation dynamics of each analyte more fully.

Interestingly we observed that the sweat and serum correlation plots of CRP were stronger than IL-6. Similarly, when classifying inflamed (subjects with active IBD) versus uninflamed (healthy cohort) individuals, CRP better differentiated these cohorts. As shown in Fig. 6, there is only a significant difference in the sweat measurements of CRP between these two groups. One possible explanation for this is that subjects with IBD are presented to the hospital in the setting of an IBD exacerbation. IL-6, however, has also been shown to be an early indicator of inflammation and has been reported to stimulate hepatocytes to rapidly produce CRP during inflammation (Darlington et al., 1986). Therefore IL-6 levels in sweat may have returned to normal levels by the time a subject’s flare worsened enough to prompt hospitalization. Alternatively, an increase in serum IL-6 may not necessarily translate into significant a rise in sweat IL-6, resulting in the non-significant difference between the active IBD group and control group. Lastly, the subjects with IBD included in this study were receiving immune suppressing medications as part of their treatment, which may have had a disproportional effect on IL-6 production, thereby mitigating any potential rise in this biomarker. Thus, CRP is a better candidate for prospective monitoring of IBD via non-invasive sweat assessments.

Our findings highlight a new technology that may be able to improve disease monitoring. This analyte platform is modifiable, enabling the measurement of up to 4 analytes at one time and allowing the evaluation of new markers or combinations of markers that may be important in disease monitoring or the study of IBD. Furthermore, the ability to have a near real time assessment of analytes allows temporal changes to be explored in a manner not possible in serum. This pilot study demonstrates the feasibility of sweat cytokine and inflammatory marker monitoring in patients with IBD. Evaluation of other potentially relevant markers in IBD are underway in larger patient populations in hopes of delineating which markers or combination of markers may be clinically relevant.

There are several limitations to our study. First, this pilot study included a small number of subjects. While 16 subjects were recruited in the IBD cohort, some subjects did not have labs drawn each day or have sweat readings that overlapped with the time that serum was drawn, precluding their inclusion in each analysis. Additionally, each subject only had one serum sample drawn each day, providing only a single serum timepoint to relate to near-continuous sweat measurements. While our results demonstrate that a relationship exists between sweat and serum readings, additional serum readings would have provided added points for analysis. The current models being used for the sweat measurements would also need to be trained on larger more heterogeneous datasets to improve its ability to quantify and classify sweat samples. Lastly, this study was performed on hospitalized patients. Further studies are needed to see if the same observations are evident in subjects going about their daily lives outside of the hospital and to explore applications such as the prediction of flares in the outpatient setting or identification of transitions from flare to remission.

## 5. Conclusion

In summary, this work demonstrates the feasibility of tracking inflammatory biomarkers continually and non-invasively in the sweat of subjects with IBD using the wearable IBD AWARE device. We found a strong linear relationship between serum and sweat-based analytes over time and a reflection in the sweat of significant and non-significant fluctuations in serum markers. These findings demonstrate promise in the ability to remotely monitor patients with chronic diseases, including IBD, utilizing wearable and sweat-sensing technology and a significant opportunity to improve disease management. Due to the design of the IBD AWARE platform, further research can be incorporated to include other IBD-related biomarkers in sweat. Challenges that would be considered would be considering how to increase the stability of the capture probe to allow for longer use of the patches to increase the cost-effectiveness for the patient. While these results are proof of use for IBD monitoring, our work focused on hospitalized IBD patients who are sedentary, next steps would be to test the response of the IBD AWARE device on active patients with relatively higher sweat elution. Another future outlook will be to combine the continuous measurements of the biomarkers with machine learning algorithms to determine the prognosis of IBD patients in active flare-up or remission.

## Supplementary Material

1

## Figures and Tables

**Table 1:** Baseline characteristics of participants at enrollment.

	Cohort, n = 16 (%)
Age, years, median	41.5
Sex	
Female	10 (62.5)
Male	6 (37.5)
Race	
White	7 (43.75)
Black	5 (31.25)
Asian	2 (12.5)
Prefer not to say	2 (12.5)
Ethnicity	
Hispanic	1 (6.25)
Not Hispanic	14 (87.5)
Prefer not to say	1 (6.25)
Smoking History	
Current	3 (18.75)
Former	3 (18.75)
Never	10 (62.5)
Ulcerative Colitis Disease Extent	
E1	0 (0)
E2	5 (45.45)
E3	6 (37.5)
Crohn’s disease Location	
Ileum only	0 (0)
Colon only	3 (50)
Ileum and Colon	2 (33.33)
Upper Gastrointestinal Tract	1 (16.67)
Prior IBD Medications	
Mesalamine	6 (37.5)
Mercaptopurine, Azathioprine or Methotrexate	3 (18.75)
TNF-α	4 (25)
Vedolizumab	1 (6.25)
Ustekinumab	1 (6.25)
Tofacitinib	1 (6.25)
Current IBD Medications	
Mesalamine	1 (6.25)
Mercaptopurine, Azathioprine or Methotrexate	2 (12.50)
TNF-α	3 (18.75)
Vedolizumab	4 (25)
Ustekinumab	0 (0)
Tofacitinib	0 (0)

**Table 2:** The ratio and correlation between CRP and IL-6 in the serum to the sweat in participants.

Analyte Serum-Sweat Ratio	Subject Disease Type	R^2^
CRP		
352,410	Crohn’s Disease	1
408,685	Crohn’s Disease	0.75
314,959	Ulcerative colitis	0.43
291,459	Ulcerative colitis	1
259,547	Ulcerative colitis	1
331,107	Ulcerative colitis	1
399,875	Ulcerative colitis	1
329,600	Ulcerative colitis	0.364
IL-6		
35.20	Crohn’s Disease	0.47
25.45	Crohn’s Disease	1
35.86	Ulcerative colitis	0.9
44.29	Ulcerative colitis	1
40.34	Ulcerative colitis	1
36.49	Ulcerative colitis	0.25

## Data Availability

Data will be made available on request.
