# Supplementary material for: Longitudinal monitoring of IL-6 and CRP in inflammatory bowel disease using IBD-AWARE
Source: Biosens Bioelectron X. Author manuscript; Available in PMC 2024 Feb 5. (PMC10843811; doi:10.1016/j.biosx.2023.100435)
Supplement: 1 [file NIHMS1962263-supplement-1.docx]

Supplemental Table 1. Baseline characteristics of the healthy subject cohort.

|  | Cohort, n=10 (%) |
| --- | --- |
| Age, years, median | 26 |
| Sex  Female  Male | 3 (30)  7 (70) |
| Race |  |
| White | 4 (40) |
| Black | 1 (10) |
| Asian | 5 (50) |
| Ethnicity |  |
| Not Hispanic | 10 (100) |
|  |  |

Supplemental Table 2. Serum and sweat measurements for CRP and IL-6 for subjects with two or more serum measurements.

|  |  |  | CRP (pg/ml) | | IL-6 (pg/ml) | |
| --- | --- | --- | --- | --- | --- | --- |
| Participant | Disease | Day | Sweat | Serum | Sweat | Serum |
| 2 | CD | 1 | 1,013.59758 | 104,600,000 | 2.79841667 | 31.66 |
|  |  | 2 | --- | --- | --- | --- |
|  |  | 3 | 947.104 | 55,500,000 | 3.137916667 | 24.33 |
| 4 | CD | 1 | 983.0995 | 16,300,000 | 2.7015 | 2.08 |
|  |  | 2 | 1,001.9372 | 11,000,000 | 3.004333333 | --- |
|  |  | 3 | 1,004.43483 | 8,600,000 | 2.804583333 | 6.43 |
| 5 | UC | 1 | 991.90 | 7,700,000 | 1.8434 | 3.67 |
|  |  | 2 | 966.80 | 4,000,000 | 2.014286 | 8.79 |
|  |  | 3 | 953.76 | 5,800,000 | 2.285172 | 11.33 |
| 6 | UC | 1 | --- | 10,100,000 | --- | 1.68 |
|  |  | 2 | --- | 58,00,000 | --- | --- |
|  |  | 3  4 | 1,137.697  900.569434 | 3,800,000  2,200,000 | 2.376087  2.243113 | ---  0.68 |
| 7 | CD | 1 | 1,002.451 | 99,900,000 | 1.677541 | 4.46 |
|  |  | 2 | 895.6306 | 97,200,000 | 2.197417 | 4.2 |
|  |  | 3  4 | 927.2134  941.3254 | 121,400,000  116,000,000 | 2.282528  2.183916 | 5.58  16.31 |
| 8 | UC | 1 | 974.5689 | 6,700,000 | 2.036 | --- |
|  |  | 2 | 525.1346 | 3,500,000 | 0.449167 | 1.4 |
| 10 | UC | 1 | 941.8021 | 17,800,000 | 2.447321 | 2.11 |
|  |  | 2 | --- | --- | --- | 4.2 |
|  |  | 3  4 | 1,035 .955  1,025.9066 | 19,100,000  44,400,000 | 1.725417  2.02725 | 3.86  24.19 |
| 12 | UC | 1  2 | 1,015.0255  1,175.3822 | 86,900,000  76,300,000 | 2.1271  1.9036 | ---  1.4 |
| 14 | UC | 1  2  3 | 930.6426  972.5266  910.1638 | 6,000,000  3,200,000  4,400,000 | 2.1  1.91  1.81 | ---  1.8  2.9 |
| 15 | UC | 1 | 923.6715 | 127,800,000 | 2.36025 | --- |
|  | UC | 1 | 919.4272 | 79100000 | 2.21766 | 2.0 |
| 16 | UC | 1 | 948.2 | 96,800,000 | 2.1 | 19.7 |
|  | UC | 2 | 961.6 | 45,978,000 | 2.2 | 18.2 |
|  | UC | 3 | 1,002.0 | 37,913,100 | 2.8 | 31.1 |
|  | UC | 4 | 960.0 | 37,300,000 | 2.5 | 10.0 |

Supplemental Table 3. Significance of the change in serum and sweat concentrations over time. Analysis of various time periods for each sweat analyte around the serum point estimate was performed.

|  | Serum Analytes Over Time | Sweat Analytes Over Time | | | |
| --- | --- | --- | --- | --- | --- |
|  |  | -2 Hour | +/-20 Minute | +/-10 Minute | +/-5 Minute |
| CRP | p=0.024 | p=0.41 | p=0.47 | p=0.47 | p=0.42 |
| IL-6 | p=0.148 | p=0.04 | p=0.03 | p=0.03 | p=0.03 |


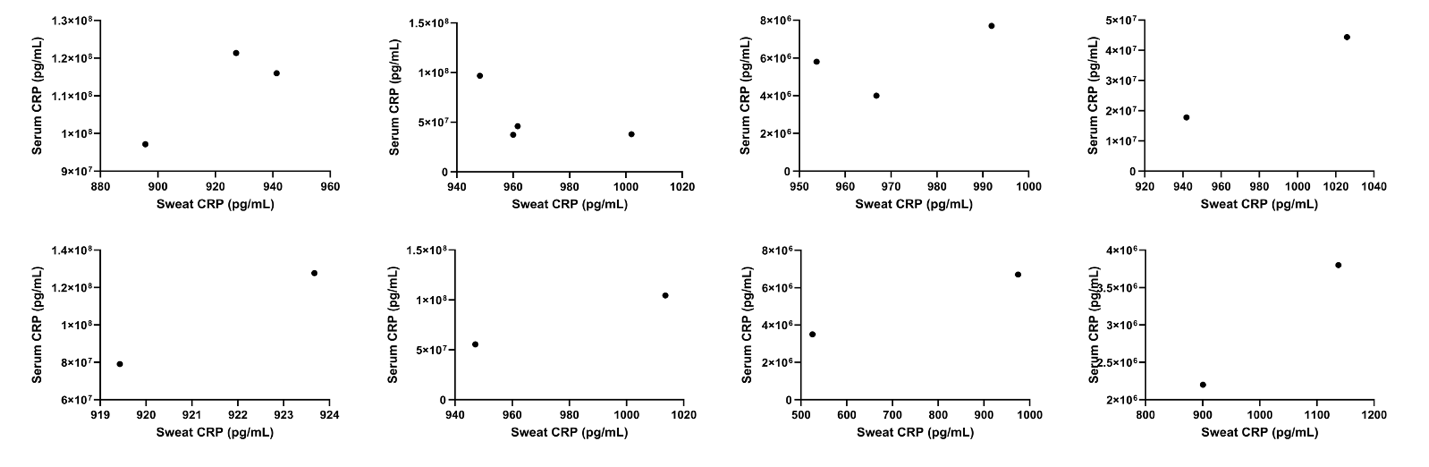


Supplemental Figure 1. The serum-to-sweat ratio for CRP in the eight subjects with two or more days of overlapping serum and sweat readings.


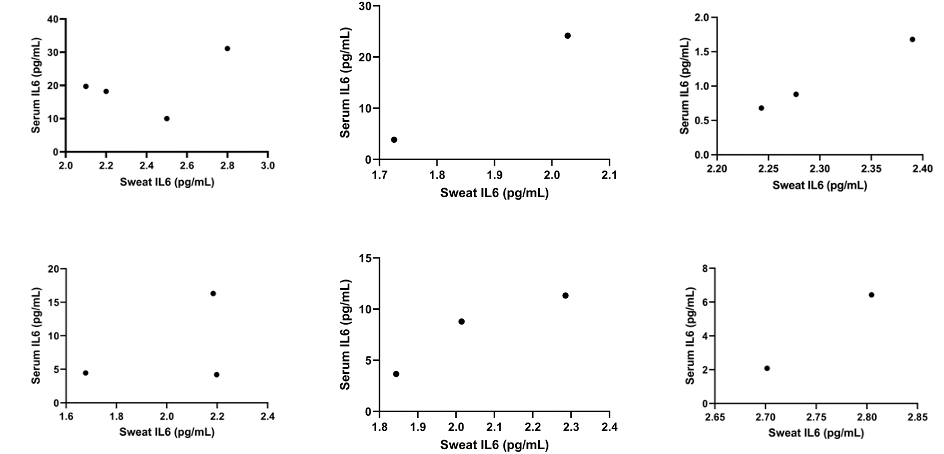


Supplemental Figure 2. The serum-to-sweat ratio for IL-6 in the six subjects with two or more days of overlapping serum and sweat readings.


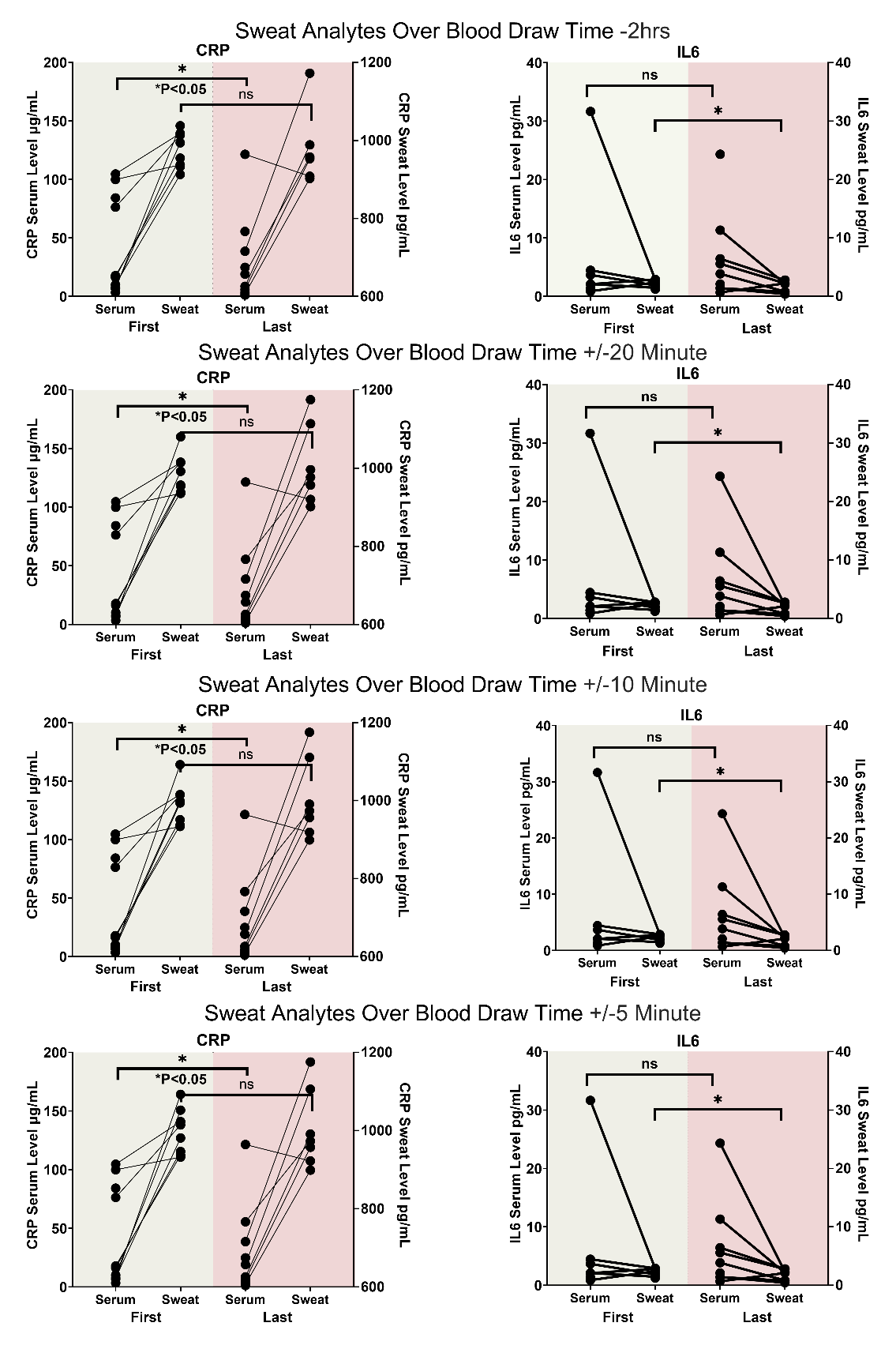


Supplemental Figure 3. Changes in serum and sweat analytes between the first and last day of enrollment at varying time periods around the serum point estimate.
